# Multi-platform integration of histopathological images and omics data predicts molecular features and prognosis of hepatocellular carcinoma

**DOI:** 10.3389/fonc.2025.1591165

**Published:** 2025-07-22

**Authors:** Linyan Chen, Yang Li, Zhiyuan Zhang, Tongshu Yang, Hao Zeng

**Affiliations:** ^1^ Department of Biotherapy, Cancer Center and State Key Laboratory of Biotherapy, West China Hospital, Sichuan University, Chengdu, China; ^2^ Division of Gastrointestinginal Surgery Ward, Department of General Surgery, West China Hospital, Sichuan University, Chengdu, China

**Keywords:** liver cancer, histopathology, genomics, transcriptomics, proteomics

## Abstract

**Background:**

Computer-aided histopathological image analysis is increasingly used for image evaluation and decision-making in cancer patients. This study extracted quantitative histopathological image features to predict molecular features, and combined them with omics data to predict prognosis of hepatocellular carcinoma (HCC) patients.

**Methods:**

Totally 334 patients from The Cancer Genome Atlas were divided equally into the training and testing sets. Histopathological image features and multiple omics data (somatic mutation, mRNA expression, and protein expression) were used alone or in combination to build prediction models through machine learning. Areas under receiver operating characteristic curves (AUCs) were assessed for 1-year, 3-year, and 5-year overall survival (OS).

**Results:**

Histopathological image features were able to predict somatic mutations: *TERT* promoter (AUC = 0.926), *TP53* (AUC = 0.893), *CTNNB1* (AUC = 0.885), *ALB* (AUC = 0.879), molecular subtypes (AUCs from 0.905 to 0.932), and OS (5-year AUC = 0.819) in the testing set, which also had good performances for OS in the external validation sets of tissue microarrays from 263 patients (5-year AUCs from 0.682 to 0.761). Furthermore, the integrated models of histopathological image features and omics data increased the accuracy of prognosis prediction, especially the multi-platform model that combined all types of features (5-year AUC = 0.904). The risk score based on the multi-platform model was a significant predictor for OS in the testing set (HR = 15.09, *p* < 0.0001). Additionally, the multi-platform model achieved a higher net benefit in decision curve analysis.

**Conclusion:**

Histopathological image features had the potential to predict molecular features and survival outcomes, and could be integrated with multiple omics data as a practical tool for prognosis prediction and risk stratification, facilitating personalized medicine for HCC patients.

## Introduction

Liver cancer is the sixth most commonly diagnosed cancer and ranks third in cancer-related death worldwide (1). Liver cancer has exacted a heavy disease burden due to its high incidence and mortality. In the United States, the incidence rate of liver cancer increased by 1.3% annually between 2012 and 2021, and the mortality rate increased at an average annual rate of 0.3% from 2013 through 2022 (2). Primary liver cancer consists of hepatocellular carcinoma (HCC) (75-85%), intrahepatic cholangiocarcinoma (10-15%), and other rare tumors (3). HCC typically develops from chronic liver disease, with main risk factors including hepatitis B virus (HBV) or hepatitis C virus (HCV) infection, alcohol abuse, diabetes, and nonalcoholic fatty liver disease (4). Considering that HCC is a heterogeneous group of disorders, the knowledge about molecular signatures and personalized medicine has continued to develop in recent years (5).

Comprehensive genome and transcriptome characterization of HCC has shown that HCC is highly heterogeneous at the molecular level (6, 7). Somatic mutations in the *TERT* promoter (observed in 44% of HCC and encoding for the catalytic subunit of telomerase), *TP53* (31%, regulating the cell cycle), *CTNNB1* (27%, encoding β-catenin, the Wnt pathway oncogene) and *ALB* (13%, encoding albumin) were most common (8). Specific genetic alterations in HCC were associated with distinct histopathological manifestations (9, 10). For example, HCC with *CTNNB1* mutation was characterized by large size, well-differentiation, cholestasis, microtrabecular and pseudoglandular patterns, and lack of inflammatory infiltration; at the same time, *TP53* mutated HCC exhibited features such as compact and poor-differentiated tumors, multinuclear and polymorphous cells, macrovascular and microvascular invasion (10). Moreover, unsupervised clustering of copy number alteration, mRNA and miRNA expression, DNA methylation, and protein level obtained three integrated molecular subtypes related to the demographic, pathological, and molecular features of HCC patients (8). By comparison, the subtype1 tumors had higher histological grades, more macrovascular invasion, fewer mutations of *TERT* promoter and *CTNNB1*, and a significantly worse prognosis (8). Overall, these integrated analyses emphasized the molecular diversity of HCC and the association of carcinogenic mechanisms with histopathological patterns.

Histopathological images of tumors have extremely high magnification, making it difficult to perform an exhaustive visual inspection. Recently, computer-aided image analysis systems and artificial intelligence have been rapidly developed to recognize subtle image features and assist clinicians in tumor classification, mutation prediction, and prognosis assessment (11–15). Yu et al. extracted image features from histopathological slides and built machine learning models to distinguish lung adenocarcinoma from squamous cell carcinoma and predict patient prognosis (11). Coudray et al. used deep learning to classify lung tumor types and predict common mutations (e.g., *STK11*, *EGFR*, *KRAS*, and *TP53*) from histopathological images (12). In addition, computer-aided histopathological image analysis has been considered valuable for predicting survival outcomes in liver cancer patients (13–15). Molecular characteristics such as mutation and gene expression were also widely adopted for prognosis prediction in cancer patients. The integration of genomics and histopathological image features could enhance the capability to predict prognosis, which has been reported in several cancers, including lung, ovarian and breast cancers (16–18). However, it remains unclear whether the integration can be applied to HCC patients.

In this study, we demonstrated a machine learning-based strategy to analyze histopathological images to achieve automatic prediction of molecular features and prognosis in HCC patients. In addition to common mutations (*TERT* promoter, *TP53*, *CTNNB1*, and *ALB*), the three molecular subtypes of HCC were also distinguished by histopathological image features. Furthermore, image features associated with survival outcomes were utilized to establish a prediction model. The prognostic value was externally validated in independent cohorts. Finally, we developed an integrated model using a combination of histopathological image features, genomics, transcriptomics, and proteomics data, which could enhance the accuracy of prognosis prediction for HCC patients.

## Materials and methods

### Histopathological image datasets

**Figure 1:**
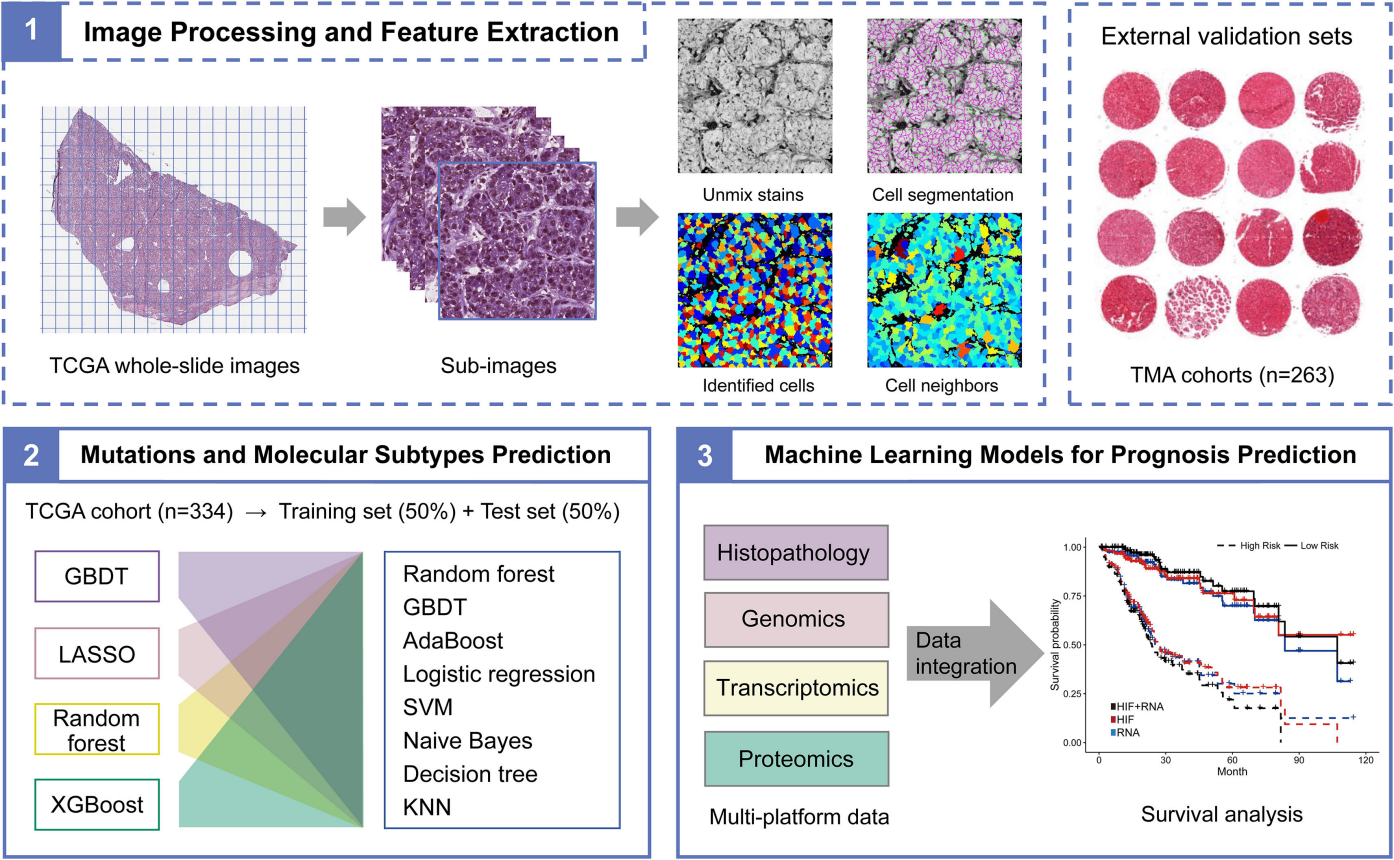
The flowchart of histopathological image processing and multi-platform data integration. (1) Whole-slide images were tiled into non-overlapping 1000×1000 pixel sub-images using Openslide-Python, and 60 sub-images were randomly selected. CellProfiler was applied to segment cells and extract histopathological image features through idenfication and measurement modules. (2) The TCGA cohort was randomly divided into training set and testing set in proportion of 1:1. Machine learning models based on histopathological image features were used to predict somatic mutations and molecular subtypes. (3) The prognostic values of models that integrated image features or multi-platform data through random forest were assessed in the testing or validation sets.

Whole-slide histopathological images and corresponding genetic data of 334 HCC patients were downloaded from The Cancer Genome Atlas (TCGA, https://portal.gdc.cancer.gov/) and The Cancer Imaging Archive (TCIA, http://www.cancerimagingarchive.net/) portals. Tissue microarray (TMA) images from resected tumors and clinical information of 263 HCC patients (HLivHCC180Sur02, HLivHCC180Sur03, and HLivH180Su14) were provided by Shanghai Outdo Biotech Co., Ltd. (Shanghai, China). Each TMA contained 90 points of 1.5 mm-diameter disk of formalin-fixed, paraffin-embedded tumor tissues from individuals aged 25-78 years, and poor-quality images were excluded. The overall flowchart of image feature extraction and multi-platform data analysis was shown in **Figure 1**, and details of the main steps were described in the following sections.

### Image processing and feature extraction

Whole-slide images (40× magnification) of the TCGA dataset were tiled into non-overlapping 1000×1000 pixel sub-images using Openslide-Python (19), and 60 sub-images were randomly chosen to represent each original image. Since TMA images had smaller sizes and lower levels of magnification, we used the adjusted image processing method for the TMA datasets, and included all regions of TMA images for feature extraction (11). Afterwards, CellProfiler was employed to separate the hematoxylin and eosin stains of sub-images by “Unmix Colors” module, then identify the nuclei and cell bodies to segment cells by “Identify Primary/Secondary Objects” modules (20). In this study, we utilized CellProfiler to extract ten aspects of 536 image features, including “ Image Area Occupied”, “Object Size Shape”, “Image Intensity”, “Object Intensity”, “Image Granularity”, “Image Quality”, “Object Neighbors”, “Object Radial Distribution”, “Correlation”, and “Texture”. Briefly, these features measured the morphology, pixel intensity distribution, texture variation, and neighbors’ relationship of entire image or cells. Features of each cell were aggregated on the sub-images by mean, median, standard deviation, and decile of the values.

### Machine learning models for mutations and subtypes prediction

We aimed to use histopathological image features to predict somatic mutations (*TERT* promoter, *TP53*, *CTNNB1*, and *ALB*) and molecular subtypes (subtype1, subtype2, and subtype3) of HCC through machine learning. The TCGA-HCC dataset was randomly divided into the training set and the testing set. Gradient boosting decision tree (GBDT) (21), least absolute shrinkage and selection operator (LASSO) (22), random forest (23), and extreme gradient boosting (XGBoost) (24) were used to eliminate redundant information and select meaningful image features on the training set. Random forest, GBDT, adaptive boosting (AdaBoost) (25), logistic regression (25), support vector machine (SVM) (26), naive Bayes (27), decision tree (28), and K-nearest neighbor (KNN) (29) were then employed to construct models on the training set. Moreover, we conducted 5-fold cross-validation to tune the model parameters and improve the robustness of models. Afterwards, we estimated the area under curve (AUC) of receiver operating characteristic (ROC) curve to show the prediction performance of final models in the independent testing set.

### Machine learning models for prognosis prediction

We investigated whether histopathological image features could predict overall survival (OS) in patients with HCC. The training set was stratified by the median value of image features and partitioned into high-value and low-value groups. The hazard ratio (HR) and 95% confidence interval (CI) were calculated by Cox regression analysis to show the prognostic values of image features. Furthermore, we established a machine learning model using a combination of histopathological image features. We applied random forest algorithm with 5-fold cross-validation to select survival-associated features and establish a prediction model in the training set. Next, the prognostic power of model was evaluated in the testing set and the TMA datasets. Time-dependent ROC curves assessed the AUC values for 1-year, 3-year, and 5-year OS. Patients were separated into high-risk and low-risk groups by the median value of risk scores generated from the model. The Kaplan-Meier estimator and Cox regression analysis were used for the comparison of survival results.

The omics data included three types: somatic mutation, mRNA expression, and protein expression. Firstly, the genomics and transcriptomics data of the training set were processed to decrease data dimensions in order to focus on the most useful attributes. Specifically, the 100 most frequent somatic mutations were selected for modelling. Besides that, we analyzed the differences in gene expression between patients with short-term (OS of 1-12 months) and long-term survival (OS ≥ 5 years), and included the top 100 differently expressed genes (DEGs) in further analysis. The Gene Ontology (GO) enrichment analysis of DEGs were performed on Metascape (http://metascape.org). Afterwards, we followed the same analysis workflow as above to build single-omics models, then developed integrated models by combining histopathological image features and omics data (histopathology + genomics, histopathology + transcriptomics, histopathology + proteomics) or multi-platform data (all types of features). The testing set was used to assess the prediction performances of these models. In addition, the net benefits of these models were estimated by decision curve analysis (30). All analyses were conducted by R v4.2.3, and *p* < 0.05 was regarded as statistically significant.

## Results

### Histopathological image features predict mutations and subtypes

**Figure 2:**
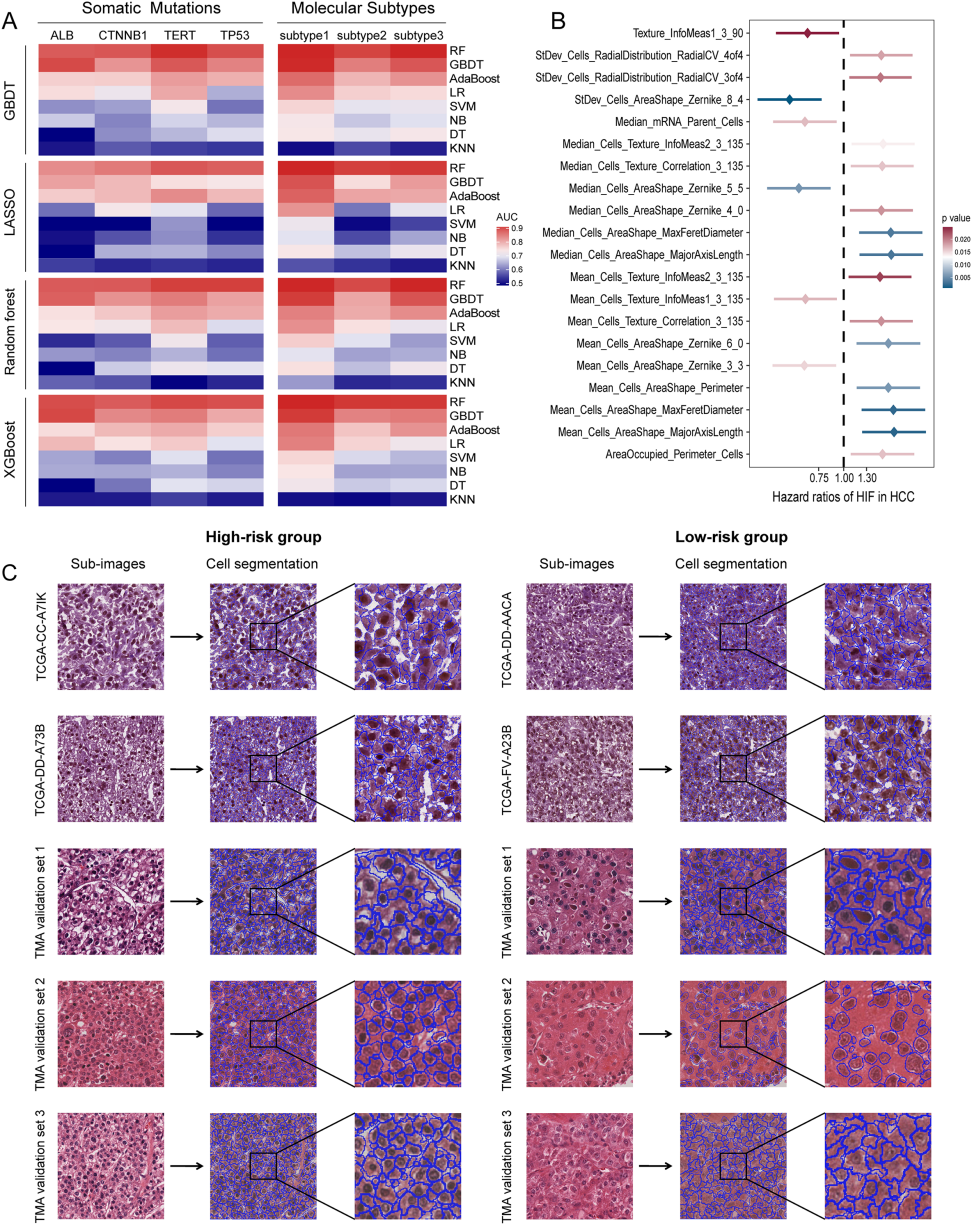
Prediction performances of histopathological image features. **(A)** GBDT, LASSO, random forest, XGBoost algorithms were used to select significative features, then random forest, GBDT, AdaBoost, logistic regression, SVM, naive Bayes, decision tree, KNN algorithms were used to build models in the training set. Five-fold cross-validation was applied to tune the model parameters and improve model robustness. The prediction accuracy for somatic mutations and molecular subtypes was evaluated by ROC curves in the testing set. **(B)** The 20 most significant histopathological image features (HIF) in univariate Cox analysis (*p* < 0.05). **(C)** Sample images of high-risk and low-risk groups. Patients were divided into high-risk and low-risk groups according to the median risk score derived from the model of histopathological image features. Cell segmentation was performed on sub-images.

To determine the association between histopathological image features and molecular signatures of HCC, we evaluated whether they could predict somatic mutations and molecular subtypes in the TCGA dataset. We randomly divided the TCGA dataset into the training set (n = 167) and the testing set (n = 167), and there was no significant difference in the patient characteristics between two groups (**Table 1**). We applied four machine learning algorithms (GBDT, LASSO, random forest, XGBoost) to select significative image features to avoid over-fitting, eight classifiers (random forest, GBDT, AdaBoost, logistic regression, SVM, naive Bayes, decision tree, KNN) with 5-fold cross-validation to build and optimize models in the training set, and then assessed their prediction performances in the testing set (**Figure 2A**). The specific AUC values for predicting mutations and subtypes by different combinations of algorithms were listed in **Supplementary Table S1**. The random forest and GBDT classifier attained good performances, and the models built by the combination of GBDT and random forest obtained higher prediction accuracy for somatic mutations: *TERT* promoter (AUC = 0.926), *TP53* (AUC = 0.893), *CTNNB1* (AUC = 0.885), *ALB* (AUC = 0.879), and molecular subtypes: subtype1 (AUC = 0.932), subtype2 (AUC = 0.905), subtype3 (AUC = 0.932) (**Supplementary Table S1**). The results indicated that histopathological image features were feasible to predict above somatic mutations and molecular subtypes of HCC through machine learning.

**Table 1:** Patient characteristics of the TCGA dataset.

Characteristics	TCGA-HCC		p
	Training set (n = 167)	Testing set (n = 167)	
Age: mean ± SD	58.4 ± 13.7	59.8 ± 12.8	0.312
Gender (%)			
Male	113 (67.7)	113 (67.7)	
Female	54 (32.3)	54 (32.3)	1.000
Cancer stage (%)			
I	80 (47.9)	81 (48.5)	
II	38 (22.8)	35 (21.0)	
III	41 (24.6)	37 (22.2)	
IV	2 (1.2)	1 (0.6)	
NA	6 (3.6)	13 (7.8)	0.517
Histological grade (%)			
G1	21 (12.6)	29 (17.4)	
G2	79 (47.3)	79 (47.3)	
G3	59 (35.3)	49 (29.3)	
G4	8 (4.8)	5 (3.0)	
NA	0 (0.0)	5 (3.0)	0.095
Survival status (%)			
Alive	111 (66.5)	107 (64.1)	
Deceased	56 (33.5)	60 (35.9)	0.646
*TERT* promoter mutation (%)			
–	49 (29.3)	42 (25.1)	
+	36 (21.6)	38 (22.8)	
NA	82 (49.1)	87 (52.1)	0.658
*TP53* mutation (%)			
–	110 (65.9)	120 (71.9)	
+	57 (34.1)	47 (28.1)	0.237
*CTNNB1* mutation (%)			
–	127 (76.0)	125 (74.9)	
+	40 (24.0)	42 (25.1)	0.799
*ALB* mutation (%)			
–	145 (86.8)	147 (88.0)	
+	22 (13.2)	20 (22.0)	0.741
Molecular subtype (%)			
Subtype1	32 (19.2)	22 (13.2)	
Subtype2	19 (11.4)	28 (16.8)	
Subtype3	29 (17.4)	23 (13.8)	
NA	87 (52.1)	94 (56.3)	0.209

### Histopathological image features predict patient prognosis

**Figure 3:**
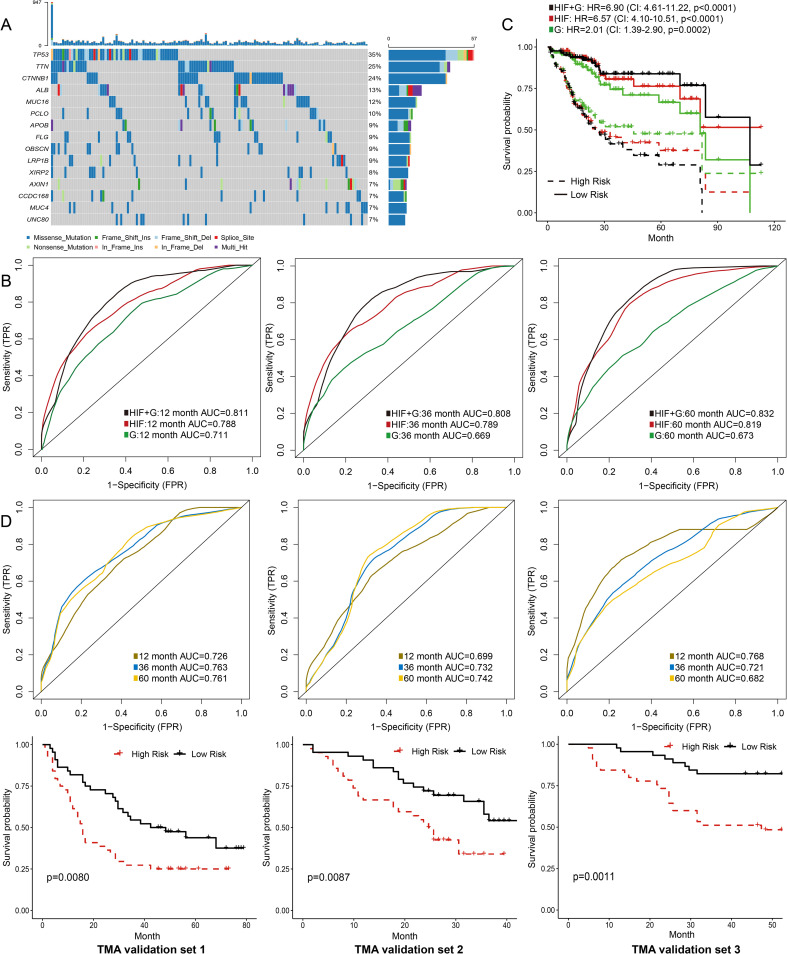
Prediction models integrating histopathological image features (HIF) and genomics. **(A)** Oncoplot of the 15 most common somatic mutations in the training set. **(B)** The ability of HIF model, genomics model, and HIF + genomics model to predict 1-year, 3-year, and 5-year survival of the testing set. The AUC values were assessed by time-dependent ROC curves. **(C)** Kaplan-Meier survival curves of high-risk and low-risk groups predicted by models in the testing set. The HR and 95% CI were calculated by Cox regression analysis. **(D)** External validation of prediction models based on histopathological image features.

To investigate the prognostic values of histopathological image features for OS, we first used univariate Cox analysis to estimate the HR between high-value and low-value groups in the training set. As shown in **Supplementary Table S2**; **Figure 2B**, the survival results of 40 image features were significantly different, and high expression of most features were adverse prognostic factors. For example, poor prognosis was relevant to higher values of Mean_Cells_AreaShape_MajorAxisLength (HR = 1.78, 95%CI: 1.24-2.57, *p* = 0.0019), Mean_Cells_AreaShape_Zernike_6_0 (HR = 1.67, 95%CI: 1.16-2.40, *p* = 0.0058), Median_Cells_Texture_Correlation_3_135 (HR = 1.56, 95%CI: 1.09-2.23, *p* = 0.0161), and StDev_Cells_Intensity_MaxIntensity (HR = 1.47, 95%CI: 1.02-2.10, *p* = 0.0372). Considering that a single feature can only reflect partial information from images, we selected informative features, built a prediction model using the random forest method, and calculated the risk scores derived from histopathological images. The model was able to predict 1-year (AUC = 0.788), 3-year (AUC = 0.789), and 5-year OS (AUC = 0.819) in the testing set (**Figure 3B**). According to the median risk score, the testing set was categorized into high-risk and low-risk groups with equal sizes. Compared to using a single feature (**Supplementary Table S2**), survival outcomes were remarkably different between high-risk group and low-risk group according to the model (HR = 6.57, 95%CI: 4.10-10.51, *p* < 0.0001; **Figure 3C**).

TMA images of 263 patients were processed as external validation sets (**Table 2**). Our model had good performances in predicting 1-year, 3-year, and 5-year OS in the validation set 1 (AUC = 0.726-0.763), the validation set 2 (AUC = 0.699-0.732), and the validation set 3 (AUC = 0.682-0.768) (**Figure 3D**). Moreover, high-risk groups in validation set 1 (*p* = 0.0080), validation set 2 (*p* = 0.0087), and validation set 3 (*p* = 0.0011) showed a significant correlation with poor prognosis (**Figure 3D**). **Figure 2C** displayed the cell segmentation process of some representative histopathological images of high-risk and low-risk patients. The quantitative measurement of images was able to help distinguish the differences of cell morphology between two groups. In summary, histopathological image features were valuable in predicting prognosis in HCC patients.

**Table 2:** Patient characteristics of the TMA datasets.

Characteristics	TMA-HCC		
	Validation set 1	Validation set 2	Validation set 3
Microarray number	HLivHCC180Sur02	HLivHCC180Sur03	HLivH180Su14
Sample size	88	85	90
Surgery time	2007.1-2009.11	2010.1-2011.9	2007.6-2008.11
Last follow-up	2013.9	2013.9	2016.2
Age: mean ± SD	53.7 ± 10.1	54.1 ± 9.3	53.4 ± 10.8
Gender (%)			
Male	76 (86.4)	70 (82.4)	80 (88.9)
Female	12 (13.6)	15 (17.6)	10 (11.1)
Cancer stage (%)			
I	11 (12.5)	6 (7.1)	63 (70.0)
II	30 (34.1)	43 (50.6)	25 (27.8)
III	40 (45.5)	29 (34.1)	2 (2.2)
IV	3 (3.4)	0 (0.0)	0 (0.0)
NA	4 (4.5)	7 (8.2)	0 (0.0)
Histological grade (%)			
G1	2 (2.3)	3 (3.5)	0 (0.0)
G1-2	3 (3.4)	14 (16.5)	4 (4.4)
G2	49 (55.7)	44 (51.8)	40 (44.4)
G2-3	18 (20.4)	14 (16.5)	22 (24.4)
G3	16 (18.2)	10 (11.8)	24 (26.7)
Survival status (%)			
Alive	30 (34.1)	42 (49.4)	45 (50.0)
Deceased	58 (65.9)	43 (50.6)	45 (50.0)

### Integrating histopathological image features and genomics to predict prognosis

We next used the same random forest method to build a prediction model from genomics data (i.e., 100 most frequent somatic mutations), and integrated both histopathological and genomics features into a prediction model (**Figure 3A**). The genomics model had lower prediction accuracy for 1-year (AUC = 0.711), 3-year (AUC = 0.669), and 5-year OS (AUC = 0.673) than the model based on histopathological image features in the testing set (**Figure 3B**). Moreover, the model integrating image features and somatic mutations improved survival prediction, with AUC of 0.811 for 1-year OS, AUC of 0.808 for 3-year OS, and AUC of 0.832 for 5-year OS. We further evaluated the risk scores of patients based on these models, and compared the survival curves between high-risk group and low-risk group (**Figure 3C**). The integrated model better distinguished low-risk patients from high-risk patients in the testing set (HR = 6.90, 95%CI: 4.61-11.22, *p* < 0.0001).

### Integrating histopathological image features and transcriptomics to predict prognosis

**Figure 4:**
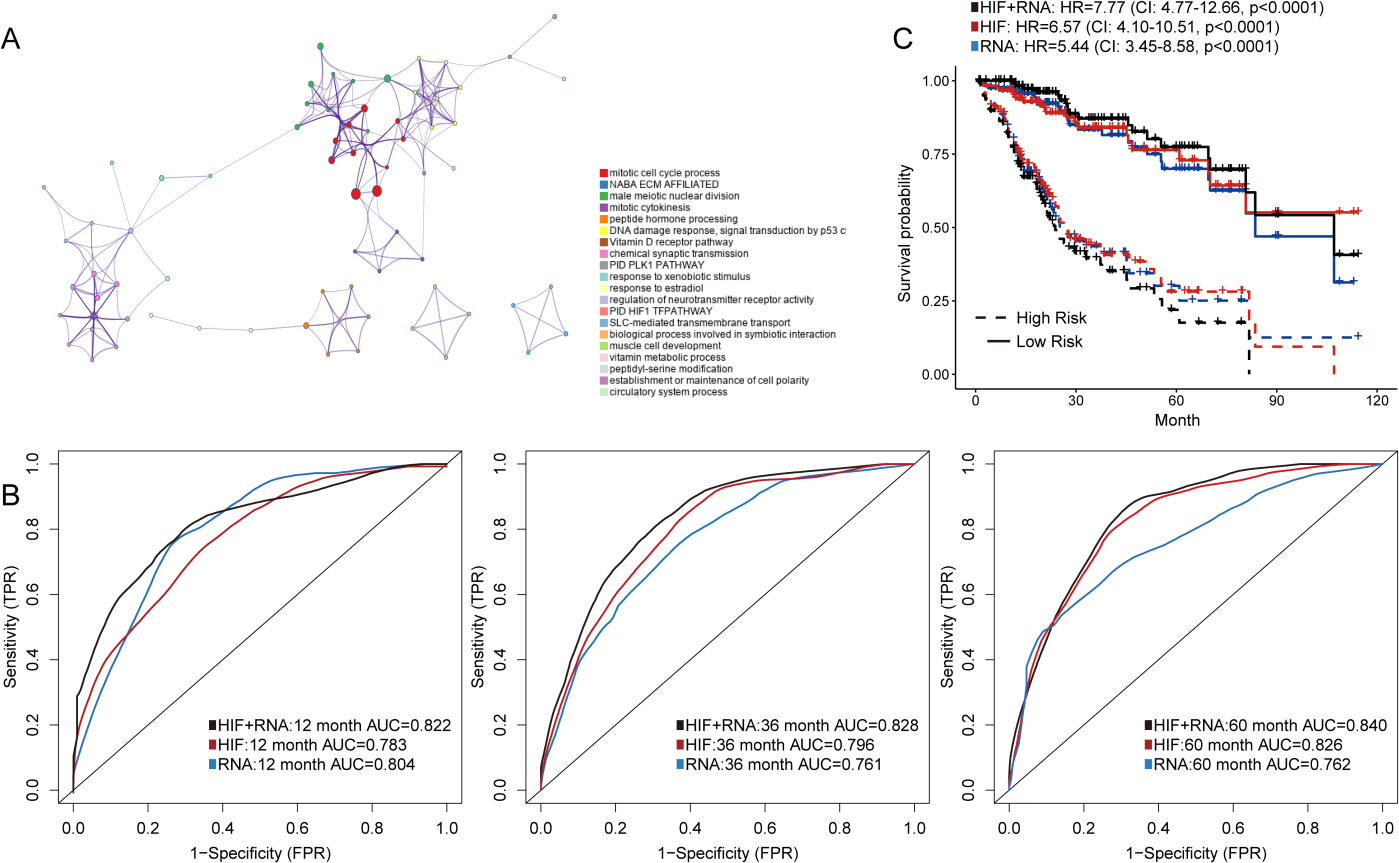
Prediction models integrating histopathological image features (HIF) and transcriptomics. **(A)** Gene Ontology (GO) enrichment analysis of differently expressed genes on Metascape. **(B)** The prediction accuracy of HIF model, transcriptomics model, and HIF + transcriptomics model in the testing set. The AUC values were evaluated by time-dependent ROC curves. **(C)** Kaplan-Meier survival curves of the testing set according to models. The HR and 95% CI were analyzed by Cox regression analysis.

For transcriptomics data, we first included the top 100 DEGs between patients with short-term (OS of 1-12 months) and long-term survival (OS ≥ 5 years) in the training set. Then we investigated the distribution of DEGs in Gene Ontology using the enrichment analysis (**Figure 4A**). The enrichment network indicated that these DEGs were mainly relevant to the functions of cell cycle process and extracellular matrix. We also established prediction models based on these gene expressions or integration of gene expressions and histopathological image features. The transcriptomics model performed equally well in predicting 1-year (AUC = 0.804) and 3-year OS (AUC = 0.761) compared with the model of histopathological image features in the testing set (**Figure 4B**). Moreover, the integrated model of image features and transcriptomics had comparably higher accuracy, with AUC values ranging from 0.822 to 0.840 (**Figure 4C**). The survival outcomes of patients with high-risk or low-risk scores were also significantly different (HR = 7.77, 95%CI: 4.77-12.66, *p* < 0.0001) in the testing set.

### Integrating histopathological image features and proteomics to predict prognosis

**Figure 5:**
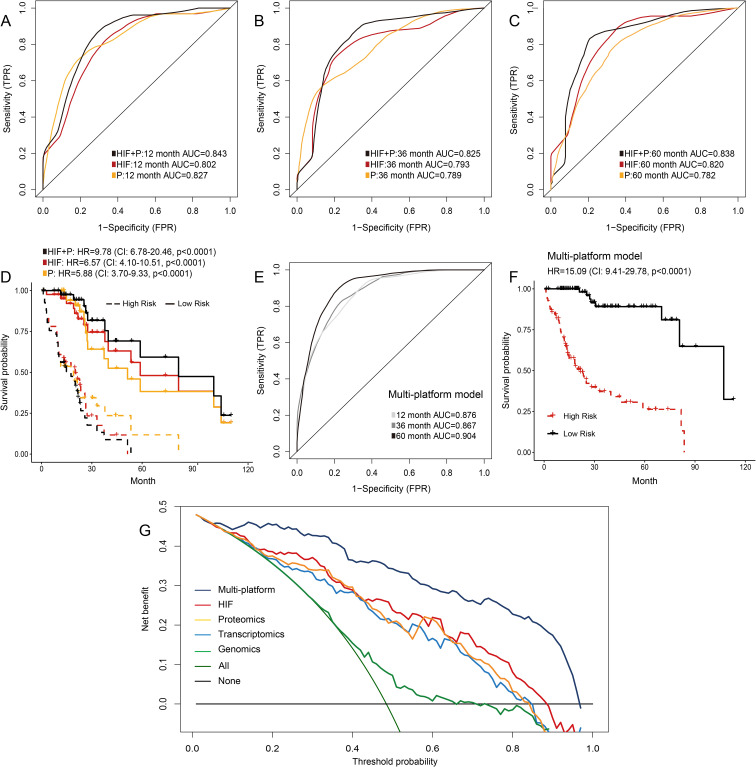
Prediction models integrating histopathological image features (HIF), proteomics, or multi-platform data. **(A-D)** Time-dependent ROC curves and Kaplan-Meier survival curves of HIF model, proteomics model, and HIF + proteomics model in the testing set. **(E, F)** Prediction performances of the model integrating multi-platform data in the testing set. **(G)** Decision curve analysis of each model. The horizontal black line represented the net benefit when no patient was treated, while the oblique green line represented the net benefit when all patients were treated. The decision curves indicated that the multi-platform model had a higher net benefit than other models across most of the threshold probability ranges.

We downloaded the expression data of 219 proteins from the TCGA dataset, which was analyzed by reverse phase protein microarray, and constructed a machine learning model to predict prognosis in HCC patients. In the testing set, the performances of the proteomics model in predicting 1-year (AUC = 0.827), 3-year (AUC = 0.789), and 5-year OS (AUC = 0.782) were comparable to that of the model using histopathological image features (**Figures 5A–C**). Furthermore, the prediction accuracy was increased when we combined image features and protein levels to predict survival outcomes (1-year AUC = 0.843, 3-year AUC = 0.825, and 5-year AUC = 0.838). In Kaplan-Meier survival curves, the integrated model obtained a more significant separation of high-risk and low-risk groups (HR = 9.78, 95%CI: 6.78-20.46, *p* < 0.0001; **Figure 5D**).

### Integrating multi-platform data to predict prognosis

In previous sections, these results suggested that combining histopathological image features and omics data could optimize modelling of predicting prognosis. Furthermore, we assessed the prognostic power of integrating multi-platform data (histopathological image features, genomics, transcriptomics, and proteomics) into a unified prediction model. The multi-platform model successfully predicted survival outcomes of the testing set, with 1-year, 3-year, and 5-year AUC values reaching 0.876, 0.867, and 0.904, respectively (**Figure 5E**). The risk score based on the multi-platform model was a significant predictor for OS in the testing set (HR = 15.09, 95%CI: 9.41-29.78, *p* < 0.0001, **Figure 5F**). Decision curve analysis can estimate the net benefits of treating patients according to these models, which demonstrated that the multi-platform model had a higher net benefit in clinical decision-making than other models using a single type of feature (**Figure 5G**).

## Discussion

Histopathological images have guiding significance for the diagnosis, grading, and prognosis of liver cancer. However, traditional visual inspection provides limited information regarding histopathological characteristics. In this study, we used quantitative features extracted from histopathological images through image analysis software to represent the morphological properties of tumor cells. We next explored the application of histopathological image features in predicting somatic mutations, molecular subtypes, and survival outcomes of HCC patients. Furthermore, we investigated whether the multi-platform integration of histopathological image features and omics data (somatic mutation, mRNA expression, and protein expression) would improve prognosis prediction. The results showed that the integrated models, especially the multi-platform model, achieved better prediction performances than the models using histopathological images or omics features alone. In summary, our study indicated that histopathological image features had the potential to predict molecular features, and could be used alone or combined with omics features to predict prognosis in HCC patients.

The driver mutations of HCC have a great impact on tumor progression and treatment options. For example, β-catenin (encoded by *CTNNB1*) is a component of the Wnt pathway, which plays an essential role in regulating tumor cell proliferation, angiogenesis, and metabolism (31). *CTNNB1* mutated HCCs were resistant to anti-PD-1 therapy due to the immune escape promoted by β-catenin activation (32). Therefore, predicting mutations from histopathological images may be beneficial for the treatments of HCC patients. Histopathological image features can be categorized into handcrafted and unsupervised features, which have their own strengths and weaknesses (33). Handcrafted features have greater interpretability and enable the measurement of specific morphological attributes. Unsupervised features generated from deep learning have broad applicability and high accuracy, but they are less intuitive and rely on large amounts of training samples. A study has trained deep learning models to predict *CTNNB1*, *FMN2*, *TP53*, and *ZFX4* mutations using HCC histopathological images (external AUCs from 0.724 to 0.898) (34). Another study showed that deep learning could identify *ALB*, *CSMD3*, *OBSCN*, *PCLO*, and *RYR2* mutations from histopathological images of HCC (external AUCs from 0.718 to 0.797) (35). In the present study, machine learning models based on handcrafted image features also displayed good performances in predicting *TERT* promoter, *TP53*, *CTNNB1*, and *ALB* mutations (AUCs from 0.879 to 0.926). In addition, we performed the prediction of molecular subtypes in HCC (AUCs from 0.905 to 0.932), which has not been reported before. These results demonstrated the rich molecular information contained in histopathological images of HCC. Moreover, our study suggested that the combination of GBDT and random forest may be more suitable for prediction modelling for this purpose among machine learning methods. However, our models lacked external validation, thus they should be further improved by large datasets with available molecular data.

Cancer patients with the same stage and pathological grade can have diverse survival outcomes (36). Recently, the automated assessment of cellular morphology in histopathological images showed significant prognostic value for HCC (14, 15). In this study, we also investigated the utilization of histopathological images for the prognosis prediction of HCC patients. In univariate analysis, the AreaShape features were associated with survival outcomes in HCC, such as MajorAxisLength, MaxFeretDiameter, and Zernike shape features. Texture features were also predictive of patient prognosis, which quantified the intensity variations in grayscale images. For example, the Correlation measures the linear dependency of intensity values, Variance describes the variation of intensity values, and InfoMeas1/2 measures the total information based on the recurring spatial relationship between specific intensity values (37). However, as the single image feature only utilized part of the image characteristics, the prediction ability was limited (38). Therefore, we developed a machine learning model based on multiple histopathological image features to improve survival prediction, which reached high accuracy in predicting short-term and long-term survival in the testing set. In addition, the histopathological image feature-based model maintained its prognostic power in three external validation sets with different patient characteristics, indicating the feasibility and generalizability of our model.

Genomics analysis has the advantage that it can provide an in-depth understanding of the potential molecular characteristics of tumors (39). Therefore, some researches have explored the combination of molecular and morphological features of tumors to improve the ability to predict prognosis (40, 41). Compared to previous study using deep learning to develop histopathology-genomics prognostic models in HCC (42), we predicted the prognosis of HCC patients by integrating handcrafted image features and more omics data, including proteomics data. Handcrafted image features have the advantage of higher interpretability and require fewer training samples than unsupervised features derived from deep learning (33). We found that the accuracy of the histopathological image feature-based model was comparable to that of the transcriptomics model and proteomics model. These findings demonstrated that histopathological image features from easily accessible and low-cost sections may provide potential prognostic information, which was of great significance for institutions with limited resources or unable to routinely perform omics testing. Furthermore, the multi-platform integrated models had better prediction performances than those models based only on histopathological images or omics information. The multi-platform integration can provide personalized risk stratification and prognostic assessment, which may ultimately facilitate refined hierarchical management and treatment selection. For instance, clinicians can use the multi-platform model to generate personalized risk scores, combined with clinical staging to predict survival outcomes and guide subsequent management. High-risk patients can be scheduled for more intensive imaging follow-up and more aggressive interventions, while low-risk patients can reduce the frequency of follow-up appropriately to alleviate their burden. However, since the TMA datasets lacked genetic data, we were unable to externally validate the multi-platform models, thus the generalizability of our approach needs further investigation.

This study also had some limitations. Firstly, our models were constructed on limited samples and omics data from the TCGA database, thus it was necessary to expand the samples and available omics data in the future. In addition, considering the potential biases inherent in retrospective analyses, the results should be validated through prospective studies. Secondly, the prediction AUCs of multi-platform model for 1-year and 3-year OS were still less than 0.9, thus further algorithm improvement is needed to enhance its prediction performance. Other endpoints such as recurrence-free survival or disease-specific survival, can also be analyzed to enhance clinical relevance. Moreover, the information regarding HBV infection, other risk factors, and adjuvant treatments were unavailable in TMA datasets, which might be confounding factors affecting survival outcomes. Futhermore, histopathological images in the TMA datasets may have potential bias, because the representative regions of tumors were more likely to be selected. Pathologists were confronted with multiple slides rather than typical pathological patterns of cases in routine work. The model was not intended to replace pathologists’ examination, but to improve the practice of pathology (43). Therefore, although the model based on histopathological image features showed potential generalizability in the TMA datasets, it still needs to be verified by whole-slide images of large-scale studies, and clinical characteristics should be considered in future studies.

In conclusion, our study demonstrated the feasibility of histopathological image features in predicting somatic mutations, molecular subtypes, and survival outcomes in HCC patients through machine learning. Furthermore, multi-platform integration of histopathological image features with omics data could be a promising modality to assist clinicians in the prognosis prediction of HCC patients. The approach may contribute to personalized medicine and be extended to other types of tumors.

## Data Availability

The raw data supporting the conclusions of this article will be made available by the authors, without undue reservation.
