# Supplementary material for: Multi-platform integration of histopathological images and omics data predicts molecular features and prognosis of hepatocellular carcinoma
Source: Front Oncol. 2025 Jul 22;15:1591165. doi: 10.3389/fonc.2025.1591165 (PMC12324167; doi:10.3389/fonc.2025.1591165)
Supplement: Supplementary file 1 [file Table1.doc]

**Table S1. Prediction of somatic mutations and molecular subtypes in the testing set.**

| **Feature**  **selection** | **Model**  **construction** | **Somatic mutations** | | | |  | **Molecular subtypes** | | |
| --- | --- | --- | --- | --- | --- | --- | --- | --- | --- |
|  |  | ***ALB*** | ***CTNNB1*** | ***TERT*** | ***TP53*** |  | **1** | **2** | **3** |
| GBDT | RF | 0.879 | 0.885 | 0.926 | 0.893 |  | 0.932 | 0.905 | 0.932 |
|  | GBDT | 0.898 | 0.822 | 0.883 | 0.840 |  | 0.929 | 0.845 | 0.884 |
|  | AdaBoost | 0.763 | 0.765 | 0.814 | 0.794 |  | 0.864 | 0.789 | 0.824 |
|  | LR | 0.750 | 0.713 | 0.802 | 0.648 |  | 0.831 | 0.763 | 0.752 |
|  | SVM | 0.622 | 0.635 | 0.726 | 0.598 |  | 0.758 | 0.700 | 0.706 |
|  | NB | 0.672 | 0.613 | 0.679 | 0.654 |  | 0.730 | 0.668 | 0.690 |
|  | DT | 0.500 | 0.620 | 0.655 | 0.687 |  | 0.736 | 0.705 | 0.731 |
|  | KNN | 0.515 | 0.576 | 0.555 | 0.547 |  | 0.508 | 0.563 | 0.524 |
| LASSO | RF | 0.806 | 0.823 | 0.856 | 0.847 |  | 0.900 | 0.878 | 0.884 |
|  | GBDT | 0.786 | 0.764 | 0.730 | 0.716 |  | 0.859 | 0.731 | 0.791 |
|  | AdaBoost | 0.747 | 0.764 | 0.806 | 0.764 |  | 0.845 | 0.783 | 0.780 |
|  | LR | 0.589 | 0.712 | 0.683 | 0.581 |  | 0.799 | 0.593 | 0.686 |
|  | SVM | 0.500 | 0.500 | 0.616 | 0.500 |  | 0.696 | 0.505 | 0.553 |
|  | NB | 0.511 | 0.565 | 0.611 | 0.539 |  | 0.680 | 0.559 | 0.629 |
|  | DT | 0.500 | 0.637 | 0.640 | 0.606 |  | 0.719 | 0.651 | 0.693 |
|  | KNN | 0.549 | 0.528 | 0.540 | 0.526 |  | 0.565 | 0.540 | 0.519 |
| RF | RF | 0.877 | 0.878 | 0.906 | 0.906 |  | 0.928 | 0.895 | 0.929 |
|  | GBDT | 0.872 | 0.822 | 0.844 | 0.806 |  | 0.909 | 0.802 | 0.885 |
|  | AdaBoost | 0.744 | 0.769 | 0.811 | 0.797 |  | 0.839 | 0.797 | 0.828 |
|  | LR | 0.738 | 0.722 | 0.781 | 0.685 |  | 0.824 | 0.744 | 0.706 |
|  | SVM | 0.535 | 0.576 | 0.718 | 0.580 |  | 0.763 | 0.690 | 0.632 |
|  | NB | 0.627 | 0.611 | 0.650 | 0.595 |  | 0.694 | 0.636 | 0.646 |
|  | DT | 0.500 | 0.671 | 0.711 | 0.696 |  | 0.745 | 0.660 | 0.717 |
|  | KNN | 0.583 | 0.570 | 0.502 | 0.530 |  | 0.629 | 0.531 | 0.539 |
| XGBoost | RF | 0.890 | 0.875 | 0.896 | 0.888 |  | 0.927 | 0.912 | 0.911 |
|  | GBDT | 0.897 | 0.831 | 0.843 | 0.798 |  | 0.910 | 0.836 | 0.845 |
|  | AdaBoost | 0.747 | 0.782 | 0.797 | 0.762 |  | 0.847 | 0.771 | 0.820 |
|  | LR | 0.782 | 0.742 | 0.766 | 0.690 |  | 0.839 | 0.756 | 0.717 |
|  | SVM | 0.638 | 0.608 | 0.688 | 0.596 |  | 0.754 | 0.682 | 0.654 |
|  | NB | 0.642 | 0.641 | 0.648 | 0.611 |  | 0.738 | 0.636 | 0.638 |
|  | DT | 0.500 | 0.599 | 0.691 | 0.677 |  | 0.719 | 0.669 | 0.682 |
|  | KNN | 0.519 | 0.519 | 0.553 | 0.520 |  | 0.510 | 0.504 | 0.516 |

Abbreviations: *ALB*, albumine; *CTNNB1*, catenin beta 1; *TERT* promoter, telomerase reverse transcriptase promoter; *TP53*, tumor protein p53; GBDT, gradient boosting decision tree; LASSO, least absolute shrinkage and selection operator; RF, random forest; XGBoost, extreme gradient boosting; AdaBoost, adaptive boosting; LR, logistic regression; SVM, support vector machine; NB, naive Bayes; DT, decision tree; KNN, K-nearest neighbor.
